# Study on the Role and Mechanism of *TOPORS* in Regulating Aortic Dissection by Mediating SUMOylation

**DOI:** 10.3390/jcdd13030110

**Published:** 2026-02-27

**Authors:** Yuan Hu, Luxi Yang, Wenjun Zhou, Hao Chen, Yuanmin Li, Bing Song, Cuntao Yu

**Affiliations:** 1 The First Clinical Medical College, Lanzhou University, Lanzhou 730099, China; huyuan3313@163.com (Y.H.); zwj-1225@163.com (W.Z.); ch1513626@163.com (H.C.); songbingldyy@163.com (B.S.); 2 Department of Cardiovascular Surgery, First Hospital of Lanzhou University, Lanzhou 730099, China; lucy_yang29@126.com (L.Y.); ldyylym@163.com (Y.L.); 3 Department of Cardiovascular Surgery, Fuwai Hospital, National Center for Cardiovascular Diseases, Chinese Academy of Medical Sciences, Peking Union Medical College, Beijing 100032, China

**Keywords:** aortic dissection, *TOPORS*, SUMOylation, VSMC, *p53*

## Abstract

Aortic dissection (AD) is a fatal acute cardiovascular emergency. SUMOylation participates in cell proliferation, apoptosis, and inflammation, but its role in AD, especially via *TOPORS*, remains unclear. This study investigates how *TOPORS* regulates AD pathogenesis through SUMOylation. AD and normal aortic samples were collected to detect *TOPORS* expression. AD mouse and VSMCs models were constructed to assess *TOPORS* depletion and overexpression effects on AD progression. In AD aortic tissues, *TOPORS* expression was upregulated, while tripartite motif containing 27 (*TRIM27*) and Sentrin-specific protease 6 (*SENP6*) expression showed no significant change. In vivo and in vitro experiments demonstrated that inhibition of *TOPORS* alleviated aortic dilation and elastic fiber degradation. *TOPORS* knockout suppressed the secretion of inflammatory cytokines (*TNF-α*, *IL-1β*, *IL-6*, and *IFN-α*), promoted *PI3K*/*AKT* phosphorylation, and downregulated *p53* signaling. The *p53* inhibitor PFTα reduced AD-induced cell apoptosis and upregulation of inflammatory cytokines. Co-immunoprecipitation further confirmed that inhibition of *TOPORS* decreases SUMOylation of *p53*. Conclusions: *TOPORS* activates *p53*, inhibits *PI3K*/*AKT* phosphorylation via SUMOylation, promotes vascular smooth muscle cell (VSMC) apoptosis and inflammation, and exacerbates AD pathogenesis.

## 1. Introduction

Aortic dissection (AD), a life-threatening acute cardiovascular disorder, is characterized by blood infiltration into the aortic media following an intimal tear, leading to separation of the true and false lumens. Its abrupt onset and rapid progression contribute to a 30% 24 h mortality rate without timely intervention, with 1-week mortality exceeding 50%, highlighting the urgency to unravel its underlying pathogenesis for targeted therapy development [1].

In recent years, significant progress has been made in identifying genetic and post-translational modification (PTM) drivers of AD. Genetic studies have implicated genes such as *CCDC80*, *ACKR1*, and *Ncf1* in AD pathogenesis: *CCDC80* maintains vascular smooth muscle cell (VSMC) contractile phenotype to protect against AD rupture [2]; *ACKR1*-expressing endothelial cells promote AD by modulating macrophage behavior [3]; and *Ncf1* knockout in VSMCs exacerbates angiotensin II-induced AD via STING pathway activation [4]. Additionally, lactylation, a novel PTM, has been linked to AD progression: mitochondrial ATP synthase subunit alpha (ATP5F1A) lactylation impairs mitochondrial function [5], while histone *H4K16* lactylation drives metabolic remodeling in VSMCs to promote aortic aneurysm and dissection [6]. These findings have advanced risk stratification and therapeutic target identification, but critical gaps remain in understanding the complex regulatory networks governing AD pathogenesis.

SUMOylation is a dynamic and reversible post-translational modification mediated by small ubiquitin-like modifiers (SUMOs). It orchestrates core cellular processes by modulating target protein activity, subcellular localization, and protein–protein interactions, including transcriptional regulation, DNA repair, cell proliferation, apoptosis, and inflammation [7,8,9,10,11]. Its molecular cascade involves SUMO precursor maturation, activation by E1 enzymes, conjugation via the sole E2 enzyme, ligation to substrates by E3 ligases, and deSUMOylation to ensure reversibility. Emerging evidence links dysregulated SUMOylation to cardiovascular pathologies [12,13]. For example, SUMOylation of transcription factor *HEY1* and fibroblast growth factor receptor 1 (*FGFR1)* modulates angiogenesis by balancing of endothelial angiogenic signals [14,15]; SUMOylation of serum response factor (SRF) drives vascular smooth muscle cell (VSMC) phenotypic switching, promoting vascular remodeling and neointimal hyperplasia [16]. However, research on SUMOylation in AD remains extremely limited. To date, only *TRIM39*, a SUMO E3 ligas, has been reported to regulate AD progression through SUMOylation of estrogen receptor alpha (ESR1) [17]. This scarcity of data indicates that the SUMOylation regulatory network in AD pathogenesis is largely uncharacterized.

The *p53* and *PI3K-AKT* pathways are well-characterized regulators of VSMC survival, proliferation, and phenotypic transition—key events in AD progression. Notably, both pathways are tightly modulated by SUMOylation: UBC9-mediated *p53* SUMOylation drives cellular senescence and exacerbates tissue injury [18]; SUMOylation of the class IA *PI3K/AKT* pathway affects cell-cycle dynamics and anti-apoptotic signaling [19]. Given the critical roles of *p53* and *PI3K-AKT* in AD, their SUMOylation status is likely a key determinant of AD pathogenesis, yet the upstream regulators of this process in the aortic wall remain unknown.

TOPORS (topoisomerase I-binding RING finger protein) is a dual-function enzyme with both SUMO E3 ligase and ubiquitin E3 ligase activity, belonging to the SUMO-targeted ubiquitin ligase (STUbL) family. Emerging studies have established its role in regulating *p53* stability and transcriptional activity through SUMOylation [20,21], with implications in tumorigenesis and neurodegenerative diseases. However, its functional relevance in cardiovascular pathologies, particularly aortic wall homeostasis, remains entirely unexplored. This represents a critical knowledge gap given the established role of SUMOylation in vascular smooth muscle cell (VSMC) phenotypic switching and extracellular matrix remodeling—core processes in aortic dissection (AD) pathogenesis. Against this backdrop, the present study focuses on *TOPORS*-mediated SUMOylation, aiming to systematically dissect its molecular mechanisms in AD pathogenesis, unravel the SUMOylation regulatory network in AD, and provide a theoretical basis for developing novel AD therapeutic targets.

## 2. Materials and Methods

### 2.1. Clinical Samples

The processing of human samples strictly adhered to the experimental protocol approved by the Ethics Committee of the First Hospital of Lanzhou University (Ethics Approval No.: LDYYLL2025-33). All participants provided written informed consent, and the entire study complied with the ethical requirements of the Declaration of Helsinki. Intraoperatively resected ascending aortic wall tissues were collected from 10 patients diagnosed with aortic dissection (AD) (5 males and 5 females). For the control group, normal ascending aortic tissues were obtained from 10 control patients without aortic dissection (5 males and 5 females), including those who underwent concurrent heart transplantation or aortic valve replacement with confirmed normal aortic diameter. Within 30 min post-explantation, all tissue samples were rinsed with pre-chilled PBS to eliminate residual blood, promptly snap-frozen in liquid nitrogen, and then stored at −80 °C for subsequent experimental procedures.

### 2.2. Mouse AD Model

Three-week-old male C57BL/6J mice, weighing 10 to 15 g, were acquired from SPF (Beijing) Biotechnology Co., Ltd. (Beijing, China). The experiment utilized a total of 40 mice. All mice were kept in a specific pathogen-free (SPF) barrier facility with a 12 h light/dark cycle, temperature ranging from 22 to 24 °C, and humidity between 50% and 70%. Animal experiments strictly adhered to the guidelines of the Animal Ethics Committee of Hebei Kangtai Medical Laboratory Service Co., Ltd. (Approval No.: MDL2024-07-03-01, Langfang, China) and complied with the Guide for the Care and Use of Laboratory Animals by the National Institutes of Health. Before surgery, the mice were randomly divided into four groups (10 mice in each group): sham operation (sham), AD model (model), sham-sh*TOPORS*, and model-sh*TOPORS*. The mouse AD model was induced by β-aminopropionitrile (BAPN): Alzet osmotic pumps (Alzet 2004, Cupertino, CA, USA) containing BAPN (1 g/kg/24 h, Sigma, Burlington, MA, USA) were implanted in the mice for continuous administration over 28 days. To ensure consistency and minimize variability, surgical procedures and drug administrations were carried out by the same experienced personnel. A small animal echocardiograph (D6LAB, VINNO, Suzhou, China) was employed to measure the diameters of the thoracic aortic root, ascending aorta, aortic arch, descending aorta, and abdominal aorta. At the experimental endpoint, mice were euthanized under deep anesthesia; aortic tissues were dissected, fixed in 4% paraformaldehyde for 24 h, and embedded in paraffin for 5 μm sectioning. All surgical interventions were performed under deep anesthesia induced by intraperitoneal injection of sodium pentobarbital (1% *w*/*v* in sterile saline, 50 mg/kg body weight). Anesthesia depth was confirmed by the absence of corneal reflex and a negative response to the toe pinch test.

### 2.3. Cell Culture and In Vitro Model

Human WSMCs were purchased from Wuhan Procell Life Science & Technology Co., Ltd. (Wuhan, China) and cultured in Smooth Muscle Cell Medium (Sciencell, San Diego, CA, USA) supplemented with 1% penicillin-streptomycin mixture. Cells were maintained in a humidified incubator at 37 °C with 5% CO_2_. To ensure cell purity and authenticity, routine mycoplasma detection and short tandem repeat (STR) profiling were performed, and no mycoplasma contamination was observed in any cell cultures.

For experimental grouping (*n* = 3), VSMCs were divided into eight groups: control group transfected with negative control siRNA (ctrl + siNC), AD model group transfected with negative control siRNA (model + siNC), control group transfected with negative control overexpression vector (ctrl + oeNC), AD model group transfected with negative control overexpression vector (model + oeNC), control group transfected with *TOPORS* siRNA (ctrl + si*TOPORS*), AD model group transfected with *TOPORS* siRNA (model + si*TOPORS*), control group transfected with *TOPORS* overexpression vector (ctrl + oe*TOPORS*), and AD model group transfected with *TOPORS* overexpression vector (model + oe*TOPORS*).

To establish the AD cell model, VSMCs were treated with angiotensin II (Ang II). To determine the optimal Ang II concentration for inducing VSMC phenotypic changes associated with AD, cells were exposed to different concentrations of Ang II (0, 0.5, 1.0, 1.5, and 2.0 μmol/L) for 48 h, followed by detection of calponin and SM22α expression levels via qRT-PCR.

For genetic manipulation, the *TOPORS* overexpression vector was constructed by synthesizing mouse *TOPORS* cDNA (Gene ID: 10210) and subcloning it into the pLVX plasmid vector (General Biosystems, Anhui, China). Small interfering RNAs (siRNAs) targeting *TOPORS* (sequences: 5′-CAUUGUGAUUCUAGUACAATT-3′; 5′-CGAUUUCGCUACCGUACAATT-3′; 5′-CUGUCUAGUAACAGAUCAATT-3′) and negative control siRNA (sequence: 5′-UUCUCCGAACGUGUCACGUTT-3′) were designed and synthesized by RiboBio Co., Ltd. (Guangzhou, China). Transfections were performed using Lipofectamine 2000 reagent (L3000015, Invitrogen, Carlsbad, CA, USA) according to the manufacturer’s instructions.

Furthermore, to investigate the regulatory role of the *p53* signaling pathway in AD progression, VSMCs were treated with the *p53* inhibitor PFTα at a final concentration of 10 μM.

### 2.4. Immunohistochemistry (IHC)

Aortic tissues from humans and mice, stored at −80 °C, were embedded in optimal cutting temperature (OCT) compound (C0171A, Beyotime, Shanghai, China) to maintain morphology. Using a cryostat microtome (CM1950, Leica, Walldorf, Germany), they were sectioned into 4 μm slices, mounted on poly-L-lysine-coated slides, and air-dried. The sections were fixed with 4% paraformaldehyde for 10 min, rinsed with PBS, and blocked with 0.3% Triton X-100 (containing 5% BSA) for 1 h at room temperature. Primary antibodies (Affinity, Cincinnati, OH, USA) were incubated overnight (12–16 h) at 4 °C: SENP6 (AF0277, 1:500), TOPORS (DF2475, 1:500), TRIM27 (DF12782, 1:500), AKT (AF0836, 1:500), p-AKT (AF0016, 1:500), p-PI3K (AF3242, 1:500), PI3K (AF6241, 1:500), and p53 (AF0879, 1:500). These primary antibodies are suitable for both human and mouse tissues. After washing with PBS, the sections were incubated with HRP-conjugated goat anti-rabbit secondary antibody (S0001, 1:3000, Affinity) in 5% BSA for 30 min at room temperature. The sections were stained with DAB (C1005, Beyotime, Shanghai, China) for 5–8 min, counterstained with hematoxylin for 30 s, dehydrated in graded ethanol, cleared in xylene, and mounted with neutral balsam. Images were captured using a light microscope (DM3000, Leica, Wetzlar, Germany) and analyzed using Image-Pro Plus 6.0; mean optical density (IOD/Area) was quantified from 5 fields per group.

### 2.5. Hematoxylin-Eosin (HE) Staining

Paraffin sections of mouse aortic tissues were dewaxed in xylene, then rehydrated through graded ethanol (100%, 95%, 80%, 70%, 2 min each) to distilled water. They were stained with hematoxylin solution from an HE Stain Kit (G1120, Solarbio, Beijing, China) for 10 min at room temperature, rinsed with tap water to remove excess stain, differentiated with kit-provided differentiating solution for 60 s, then rinsed with tap water for 15 min to blue nuclei. They were then stained with eosin solution from the same kit for 2 min, dehydrated through graded ethanol (70%, 80%, 95%, 100%, 2 min each), cleared in xylene (twice, 5 min each), mounted with neutral balsam, air-dried, and observed under a light microscope.

### 2.6. Elastica Van Gieson (EVG) Staining

Dewaxed sections were stained with Verhoeff staining solution (DC0057, Leagene, Beijing, China) for 30 min, rinsed with distilled water, differentiated with differentiating solution until elastic fibers turned black and background gray, then rinsed under running water for 5 min. They were deiodinated with 95% ethanol twice and counterstained with Orange G for 1 min, with the excess stain blotted. They were dehydrated in absolute ethanol twice (30 s each), cleared in xylene twice (5 min each), mounted with neutral balsam, and examined microscopically after drying.

### 2.7. Quantitative Real-Time PCR (qRT-PCR)

Total RNA was extracted from aortic tissue samples and VSMCs using TRIpure Reagent (RN0102, Aidlab Biotechnologies Co., Ltd., Beijing, China). RNA concentration and purity were determined with a UNano-1000 spectrophotometer (YoMim, Hangzhou, China), and RNA integrity was verified via 1% agarose gel electrophoresis. RNA was reverse-transcribed into cDNA using ExonScript RT SuperMix (A502-02, EXONGEN, Chengdu, China). qRT-PCR amplification was performed with SYBR Select Master Mix (4472920, Invitrogen, Carlsbad, CA, USA) on a Q2000B qPCR instrument (LongGene, Hangzhou, China). β-actin served as the internal reference, and the relative expression levels of *TOPORS*, *SENP6*, *TRIM27*, *PI3K*, *AKT*, and *p53* were calculated using the comparative cycle threshold (Ct) method. The gene primer information is listed in Table 1.

### 2.8. Western Blotting

Aortic tissue samples and VSMCs were lysed with RIPA lysis buffer (P0013C, Beyotime, Shanghai, China) on ice for 30 min. After lysis, the samples were centrifuged at 12,000× *g* and 4 °C for 15 min to collect supernatants, and the protein concentration was determined using a BCA kit (P0012, Beyotime, Shanghai, China). For protein denaturation, 50 μg of protein was mixed with loading buffer and heated at 95 °C for 5 min. The denatured protein was then loaded onto 10% SDS-PAGE gels, and electrophoresis was performed at 80 V until the samples entered the separating gel, after which the voltage was adjusted to 120 V. Following electrophoresis, proteins were transferred to PVDF membranes (Millipore, Boston, MA, USA) at a constant current of 300 mA in an ice bath. The membranes were blocked with 5% non-fat milk in TBST for 1 h, then incubated with primary antibodies at 4 °C overnight, followed by three washes with TBST (10 min each). Subsequently, the membranes were incubated with secondary antibodies for 1 h and washed again. Protein bands were detected using an ECL system (ChemiScope6100, CLINX, Shanghai, China), and grayscale analysis was performed with ImageJ software (v1.8.0, NIH, Bethesda, MD, USA). The primary antibodies used were SENP6 (AF0277, 1:500), TOPORS (DF2475, 1:500), TRIM27 (DF12782, 1:500), AKT (AF0836, 1:500), p-AKT (AF0016, 1:500), p-PI3K (AF3242, 1:500), PI3K (AF6241, 1:500), and p53 (AF0879, 1:500); the secondary antibody was goat anti-rabbit IgG-HRP (S0001, 1:3000), all obtained from Affinity Biosciences.

### 2.9. Enzyme-Linked Immunosorbent Assay (ELISA)

The levels of inflammatory factors, including IFN-α, IL-6, TNF-α, and IL-1β, were measured using ELISA. Specifically, the following ELISA kits were employed: Mouse TNF-α Elisa Kit (H052-1-2), Mouse IFN-α Elisa Kit (H023-1-1), Mouse IL-6 Elisa Kit (H007-1-2), and Mouse IL-1β Elisa Kit (H002-1-2), all purchased from Nanjing Jiancheng Bioengineering Institute (Nanjing, China). All experimental procedures were performed strictly according to the manufacturer’s instructions to ensure the accuracy and reproducibility of the assay results.

### 2.10. Cell Proliferation Assay

Cell proliferation was evaluated using the Cell Counting Kit-8 (CCK-8) assay and 5-ethynyl-2′-deoxyuridine (EdU) incorporation assay. For the CCK-8 assay, VSMCs were seeded in 96-well plates at a density of 1 × 10^4^ cells/well and cultured for 12 h. Afterward, 10 µL of CCK-8 reagent (Beyotime, Shanghai, China) was added to each well, followed by incubation for 1 h. Absorbance at 450 nm was measured using a microplate reader (Model 550, BIORAD, Hercules, CA, USA), where absorbance values correlated positively with cell proliferative activity.

For the EdU assay, VSMCs were seeded in 24-well plates at 5.0 × 10^4^ cells/well, incubated with 10 µmol/L EdU solution (Beyotime, Shanghai, China) at 37 °C for 2 h, then fixed with 4% paraformaldehyde (PFA) at 4 °C for 15 min. Subsequently, 100 µL of Click reaction solution was added, and nuclei were stained with Hoechst 33,342. Fluorescent signals were visualized under a fluorescence microscope to assess cell proliferation.

### 2.11. Co-Immunoprecipitation

VSMCs were pretreated with 20 mM NEM (N-Ethylmaleimide) in medium for 20 min at 37 °C to block deSUMOylation, then lysed on ice for 30 min in RIPA buffer containing 1% SDS and 20 mM NEM. After centrifugation (14,000 rpm, 15 min, 4 °C), 500 µg supernatant protein was incubated with 2 µg anti-SUMO1 antibody (CST, Cat#4930S) at 4 °C overnight with rotation. Pre-washed Protein A/G beads (30 µL) were added for 3 h at 4 °C, then pelleted (3000 g, 5 min, 4 °C). Beads were washed 4× with NEM-containing RIPA buffer, boiled with 2× SDS loading buffer for 8 min, and subjected to SDS-PAGE. Proteins were transferred to PVDF membrane, blocked, and incubated with anti-p53 antibody (AF0879, 1:500) followed by HRP-conjugated secondary antibody (S0001, 1:3000). Signals were detected via ECL chemiluminescence.

### 2.12. Cell Apoptosis Assay

Cell apoptosis was evaluated using TUNEL staining and Annexin V/PI flow cytometry. For TUNEL staining, cells were washed twice with pre-cooled PBS to remove residual medium, fixed with 4% PFA for 15 min, and permeabilized with 0.25% Triton X-100 for 10 min. Samples were then incubated with TUNEL reagent (In Situ Cell Death Detection Kit, Beyotime, Shanghai, China) at 37 °C for 50 min, followed by nuclear staining with Hoechst 33342 for 10 min. Fluorescent signals were observed under a Leica fluorescence microscope (Wetzlar, Germany), and the apoptosis rate was calculated as the ratio of TUNEL-positive apoptotic cells to total DAPI-labeled cells.

For the Annexin V/PI assay, cells were resuspended in Binding Buffer at 1 × 10^6^ cells/mL; 100 µL cell suspension was mixed with 5 µL Annexin V/FITC in 5 mL flow tubes, incubated at room temperature for 5 min in the dark, then stained with 10 µL of 20 µg/mL propidium iodide (PI) solution. After adding 400 µL PBS, samples were immediately analyzed using a flow cytometer (BeamCyte-1026, BENM DIAG, Changzhou, China) with the Annexin V/PI Apoptosis Detection Kit (V13242, Invitrogen, Carlsbad, CA, USA).

### 2.13. Statistical Analysis

GraphPad Prism 9 (San Diego, CA, USA) was utilized for conducting statistical analyses. Student’s *t*-test was employed to analyze comparisons between two groups, and one-way ANOVA followed by Tukey’s post hoc test was used for multiple groups. Statistical significance was set at *p* < 0.05. Unless otherwise stated, data are shown as mean ± standard deviation (SD).

## 3. Results

### 3.1. TOPORS Expression Was Significantly Increased in Human AD

Human aortic dissection tissues and normal aortic tissue samples were carefully collected and subsequently divided into two distinct groups for comparative analysis: the control group, which consisted of normal aortic tissues, and the aortic dissection (AD) group, which included tissues affected by the dissection. To investigate the protein expression levels of *SENP6*, *TOPORS*, and *TRIM27*, immunohistochemistry (IHC) was employed as a reliable technique. Concurrently, qRT-PCR was utilized to measure the mRNA transcription levels of these same genes, providing a comprehensive assessment of both protein and genetic expression.

**Figure 1:**
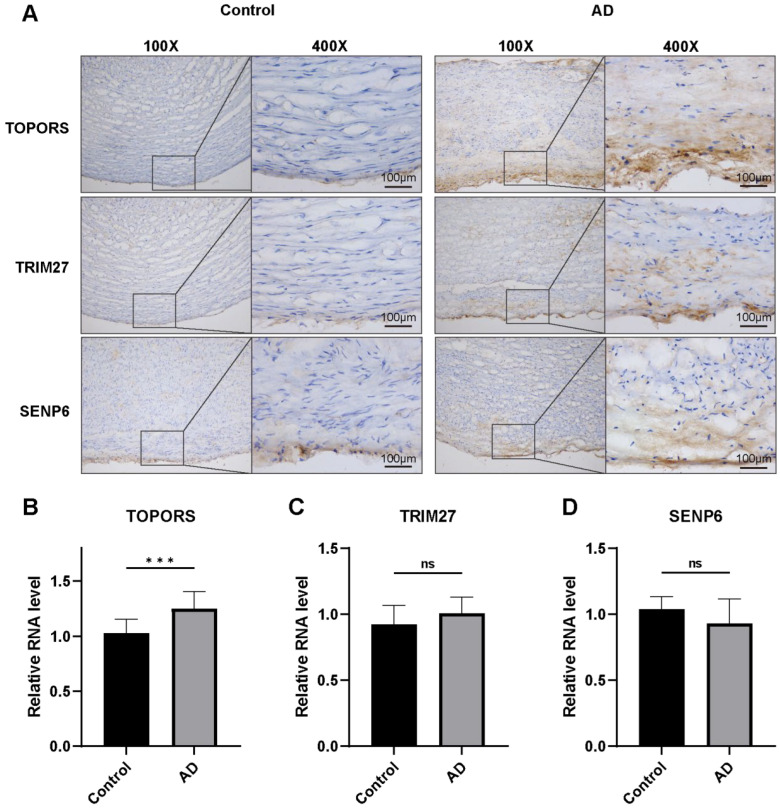
Expression levels of mRNA and protein of *TOPORS*, *TRIM27*, and SENP6 in aortic dissection (AD). (**A**) Immunohistochemistry (IHC) was used to measure the protein levels of *TOPORS*, *TRIM27*, and *SENP6* in AD (scale bar = 100 μm). Positive expression of target proteins is indicated by brown—yellow staining, while blue staining represents nuclear counterstaining with hematoxylin. (**B**–**D**) qRT-CR was employed to measure the mRNA levels of *TOPORS* (**B**), *TRIM27* (**C**), and SENP6 (**D**) in aortic dissection. (*** *p* < 0.001; ns, no significant difference; *n* = 10).

Upon conducting statistical analysis, it was observed that when compared to the control group, the protein and mRNA expression levels of *TOPORS* in the AD group exhibited a significant increase, indicating a potential role of this gene in the pathogenesis of aortic dissection (Figure 1A,B). In contrast, for *TRIM27* and *SENP6*, although there were changes in their expression levels, these alterations did not reach statistical significance, suggesting that their involvement in aortic dissection may be less pronounced or require further investigation.

### 3.2. Inhibition of TOPORS Ameliorates AD in Mice

To thoroughly investigate the pivotal role of *TOPORS* in the pathogenesis of AD, we established an AD mouse model by inducing the condition through the administration BAPN. Subsequently, we evaluated the effects of *TOPORS* knockdown on this model. All mice in the sham and sham-sh*TOPORS* groups survived, with no aortic dissection found in post-mortem examination. In the BAPN-induced model group, two mice died from ruptured aortic dissection. Of the eight surviving mice, seven developed aortic dissection, and five of them had rupture. In the model-sh*TOPORS* group, there was no mortality during the experiment. Post-mortem analysis showed six mice developed aortic dissection and three of them had rupture.

**Figure 2:**
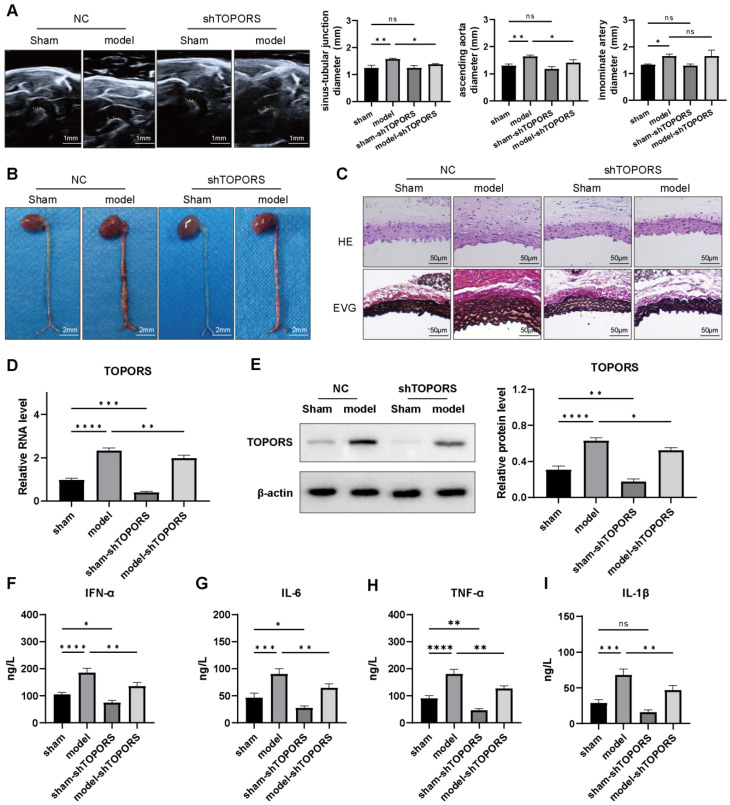
Inhibition of *TOPORS* mitigated aortic dissection (AD) in mice. (**A**): Ultrasound imaging of the mouse thoracic aorta depicting changes in aortic diameter (scale bar = 1 mm); (**B**): Gross anatomical images of the thoracic aorta demonstrating morphological variations (scale bar = 2 mm); (**C**): Representative transverse sections of aortic tissues from AD mice stained with hematoxylin and eosin (H&E) for structural analysis and elastica van Gieson (EVG) for visualization of elastic fibers; (**D**,**E**): Expression levels of *TOPORS* in aorta tissues determined by qRT-PCR for mRNA (**D**) and Western blot (**E**) for protein; F-I: Enzyme-linked immunosorbent assay (ELISA) quantification of pro-inflammatory factors in aortic tissues: (**F**) interferon-α (*IFN-*α), (**G**) interleukin-6 (*IL-6*), (**H**) tumor necrosis factor-α (*TNF-α*), and (**I**) interleukin-1β (IL-1β). Data are presented as mean ± standard deviation (SD) (*n =* 10). Significance levels are denoted as * *p* < 0.05, ** *p* < 0.01, *** *p* < 0.001, **** *p* < 0.0001, where “ns” indicates no significant difference.

Utilizing ultrasound imaging to measure aortic dilation on the 14th day post-model induction, we observed that the aortic diameter in the model group was significantly larger compared to the sham control group. However, this pronounced aortic dilation was markedly reduced in the model group treated with shRNA targeting *TOPORS* (model + sh*TOPORS* group), as depicted in Figure 2A. Gross anatomical examinations further corroborated these findings. The model group exhibited notable thoracic aortic aneurysmal enlargement, which was filled with erythrocytes, indicating severe structural damage. In stark contrast, the inhibition of *TOPORS* led to a substantial alleviation of this aortic structural deterioration, as evidenced in Figure 2B.

To delve deeper into the histopathological changes, we conducted HE and EVG staining. These analyses revealed that the AD model mice displayed a disorganized aortic architecture characterized by severe degradation of medial elastic fibers. Conversely, the knockdown of *TOPORS* preserved the integrity of the elastic fiber layers and maintained the closed circular structure of the thoracic aorta, as illustrated in Figure 2C. Molecular validation through qRT-PCR and Western blotting assays confirmed that both the mRNA and protein levels of *TOPORS* were significantly elevated in the model group. However, these levels were markedly reduced following the silencing of *TOPORS*, as shown in Figure 2D,E. Given the critical role of inflammation in the progression of AD, we performed cytokine profiling using ELISA. The results indicated that the model mice exhibited elevated levels of pro-inflammatory cytokines, including *IFN-*α, *IL-6*, *TNF-α*, IL-1β. Importantly, the inhibition of *TOPORS* significantly reversed these elevated cytokine levels, as demonstrated in Figure 2F–I. Collectively, these comprehensive data suggest that the inhibition of *TOPORS* effectively mitigates the formation of AD by restraining aortic dilation, preserving the structural integrity of the vasculature, and reducing the inflammatory responses associated with AD progression.

### 3.3. Effects of TOPORS Inhibition on PI3K/AKT and p53 Signaling Pathways

**Figure 3:**
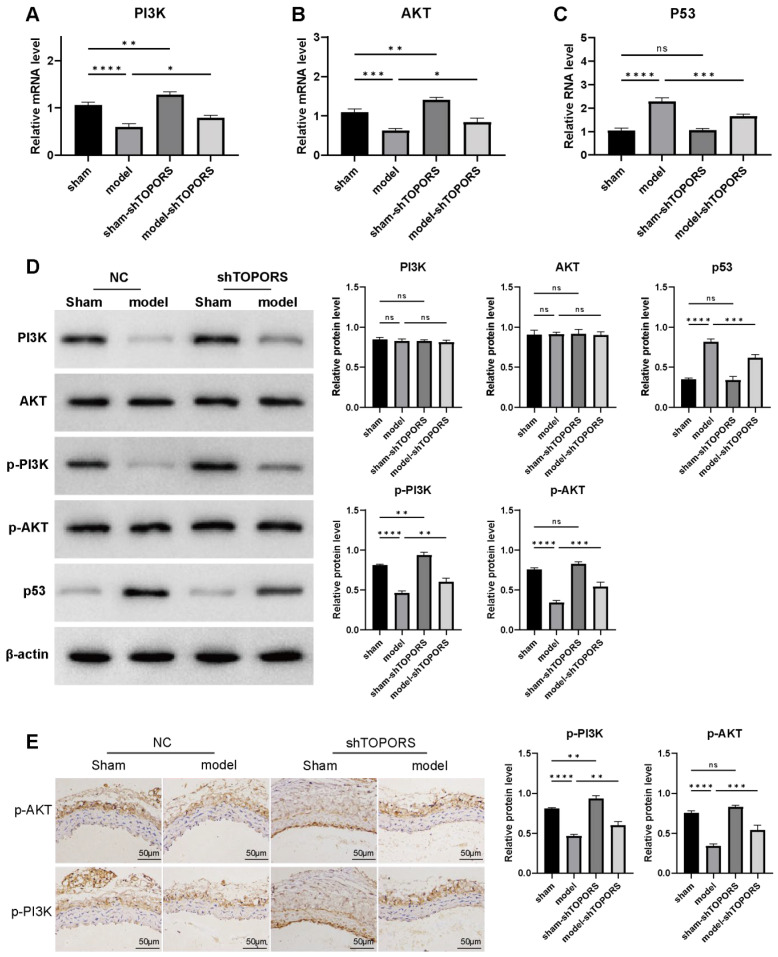
Effects of *TOPORS* inhibition on *PI3K/AKT* and *p53* signaling pathways in aortic dissection (AD) model mice. A-C: the mRNA expression profiles of *PI3K* (**A**), *AKT* (**B**), and *p53* (**C**) quantified by qRT-PCR. (**D**): Western blot analysis of protein expression, including PI3K, AKT, p-PI3K, p-AKT, and *p53*. (**E**): the spatial protein expression of p-PI3K and p-AKT in aortic tissues using immunohistochemistry (scale bar = 50 μm). Data are presented as mean ± standard deviation (SD) (*n* = 10). Significance levels are denoted as * *p* < 0.05, ** *p* < 0.01, *** *p* < 0.001, **** *p* < 0.0001, where “ns” represents no significant difference.

qRT-PCR and Western blot analyses were performed to investigate the regulatory role of *TOPORS* in signaling pathway dysregulation during AD pathogenesis. Compared with the sham group, the AD model mice exhibited significant upregulation of both mRNA and protein expression levels of *p53*. For the *PI3K/AKT* pathway, mRNA expression of *PI3K* and *AKT* was markedly decreased, whereas their protein levels remained unchanged (Figure 3A–D). Notably, the phosphorylated active forms (p-PI3K and p-AKT) showed significant downregulation (Figure 3D,E). Furthermore, targeted inhibition of *TOPORS* gene expression in the aortic dissection model partially reversed these pathological molecular alterations: *p53* upregulation was attenuated, and the reduction in p-PI3K/p-AKT levels was alleviated. These findings collectively suggest that *TOPORS* may contribute to the initiation and progression of aortic dissection by modulating the *PI3K/AKT* signaling pathway (via regulation of kinase phosphorylation) and the *p53* pathway.

### 3.4. TOPORS Regulates Aortic Dissection-like Phenotypes in VSMCs

**Figure 4:**
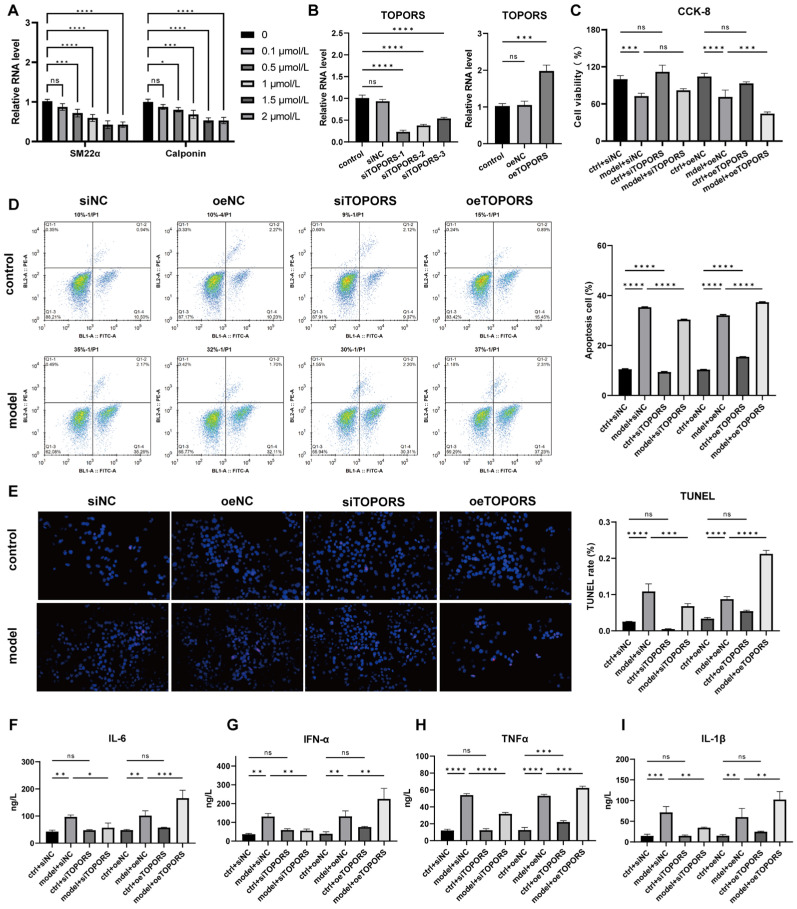
Effects of *TOPORS* on Ang-II-induced vascular smooth muscle cells (VSMCs). (**A**): Ang-II treatment leads to a dose-dependent downregulation of mRNA expression for calponin and *SM22α*, which are critical markers of the contractile VSMC phenotype; (**B**): The mRNA expression level of *TOPORS* in VSMC lines with *TOPORS* knockout (si*TOPORS*) or overexpression (oe*TOPORS*) was determined by qRT-PCR; (**C**): Regulation of *TOPORS* knockout or overexpression on the viability of Ang-II-stimulated VSMCs was assessed by CCK-8 assay; (**D**,**E**): Regulation of *TOPORS* knockout or overexpression on the apoptosis of Ang-II-stimulated VSMCs was evaluated by flow cytometry analysis (**D**) and TUNEL staining (**E**); (**F**–**I**): Regulation of *TOPORS* knockout or overexpression on the secretion of pro-inflammatory cytokines in VSMC lines were determined by ELISA: interleukin-6 (*IL-6*) (**F**), interferon-α (*IFN-*α) (**G**), tumor necrosis factor-α (*TNF-α*) (**H**), and interleukin-1β (*IL-1β*) (**I**). Data are presented as mean ± standard deviation (SD) (*n* = 3). Significance levels are denoted as * *p* < 0.05, ** *p* < 0.01, *** *p* < 0.001, **** *p* < 0.0001, where “ns” represents no significant difference.

To investigate the role of *TOPORS* in AD pathogenesis, human VSMCs were treated with Ang-II to establish an in vitro AD cell model. qRT-PCR analysis demonstrated that Ang-II dose-dependently downregulated the mRNA expression of VSMC-specific contractile genes, including *calponin* and *SM22α*, confirming the phenotypic switch of VSMCs induced by Ang-II stimulation (Figure 4A). Subsequently, *TOPORS* knockout (si*TOPORS*) and overexpression (oe*TOPORS*) VSMC lines were constructed to evaluate the impact of *TOPORS* on cell function (Figure 4B). CCK-8 assays revealed that, compared to the control group, cell viability was significantly reduced in the Ang-II-induced model group. Notably, *TOPORS* inhibition partially rescued cell viability, whereas *TOPORS* overexpression further suppressed viability (Figure 4C). Flow cytometry and TUNEL staining consistently showed that Ang-II treatment markedly increased apoptotic rates compared to controls. This pro-apoptotic effect was reversed by *TOPORS* inhibition but exacerbated by *TOPORS* overexpression (Figure 4D,E). Additionally, ELISA results indicated that Ang-II stimulation significantly elevated the secretion of pro-inflammatory cytokines, including *TNF-α*, *IL-1β*, *IL-6*, and *IFN-*α (Figure 4F–I). *TOPORS* inhibition reduced the levels of these cytokines, while *TOPORS* overexpression further promoted their release. Collectively, these data suggest that *TOPORS* may exacerbate AD-like phenotypes in VSMCs by suppressing viability, promoting apoptosis, and enhancing inflammatory responses, whereas its inhibition alleviates these pathological processes.

### 3.5. Regulation of PI3K/AKT and p53 Signaling Pathways by TOPORS in VSMCs

**Figure 5:**
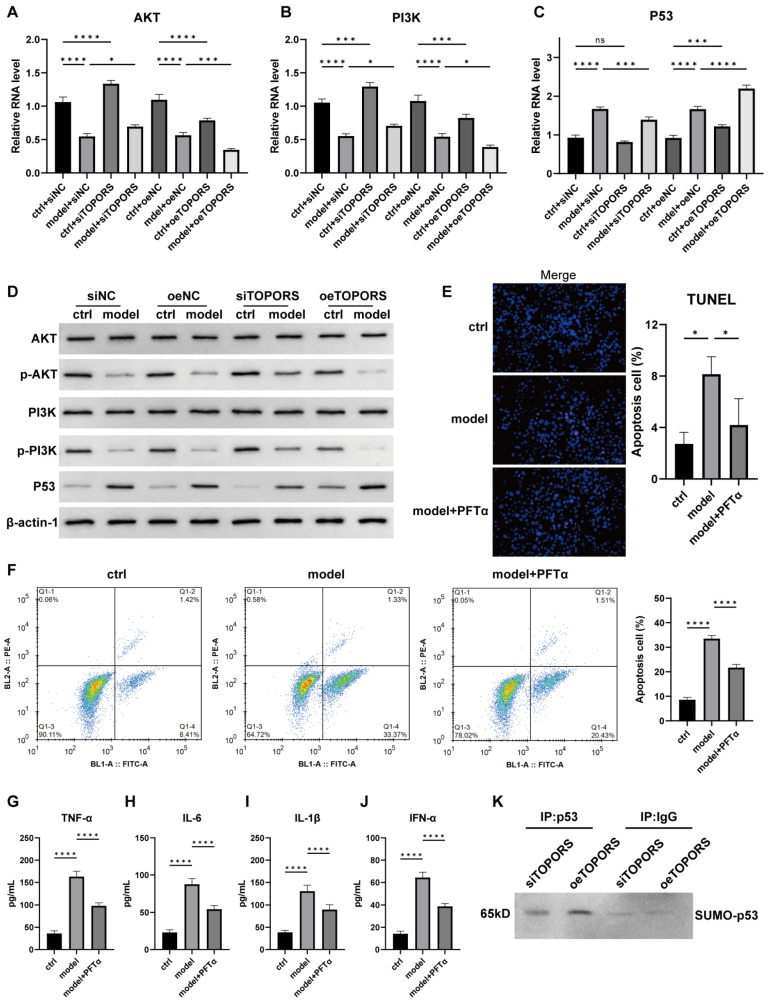
Effects of *TOPORS* on *PI3K/AKT* and *p53* signaling pathways in VSMC model of aortic dissection (**A**). (**A**–**C**): the mRNA expression profiles of *AKT* (**A**), *PI3K* (**B**), and *p53* (**C**) quantified by qRT-PCR. (**D**): Western blot analysis of protein expression, including AKT, p-AKT, PI3K, p-PI3K, and *p53*. (**E**,**F**): The apoptosis of AD model VSMC after PFT-ɑ treatment was evaluated by TUNEL staining (**E**) and flow cytometry analysis (**F**); (**G**–**J**): ELISA quantification of pro-inflammatory cytokines after PFT-ɑ treatment: tumor necrosis factor-α (*TNF-α*) (**G**), interleukin-6 (*IL-6*) (**H**), interleukin-1β (*IL-1β*) (**I**), and interferon-α (*IFN-*α) (**J**). Data are presented as mean ± standard deviation (SD) (*n* = 3). Significance levels are denoted as * *p* < 0.05, *** *p* < 0.001, **** *p* < 0.0001, where “ns” represents no significant difference. (**K**): The SUMOylation level of the *p53* gene was detected by co-immunoprecipitation (Co-IP) in the model-si*TOPORS* group and model-oe*TOPORS* group.

qRT-PCR and Western blot analyses revealed that, compared with the sham control group, the mRNA and protein expression levels of *p53* were significantly upregulated in the AD cell model group. Although the mRNA expression levels of *PI3K* and *AKT* were significantly decreased (*p* < 0.01), their protein expression levels did not change significantly, while their phosphorylation levels (p-PI3K and p-AKT) were significantly reduced (Figure 5A–D). Inhibition of *TOPORS* gene expression alleviated these changes induced by the AD model, whereas overexpression of *TOPORS* exacerbated these gene alterations. These results suggest that *TOPORS* may influence the progression of aortic dissection by regulating the *PI3K/AKT* and *p53* signaling pathways. Subsequent treatment of VSMC models with the *p53* inhibitor PFTα, followed by flow cytometry and TUNEL staining, demonstrated that PFTα could mitigate cell apoptosis induced by the AD model (Figure 5E,F) and reduce the upregulation of inflammatory factors such as *TNF-α*, *IL-1β*, *IL-6*, and *IFN-*α (Figure 5G–J). Co-immunoprecipitation experiments further confirmed that the level of SUMOylation of *p53* was significantly higher in the oe-*TOPORS* VSMC model group than in the si*TOPORS* group (Figure 5K).

## 4. Discussion

Aortic dissection (AD) remains a critical challenge in clinical and basic research due to its complex pathological mechanisms and limited therapeutic options. In recent years, SUMOylation, a key post-translational modification, has been increasingly recognized for its roles in cardiovascular diseases [14,16], yet its specific regulatory network in AD pathogenesis requires further exploration. *SENP6*, *TRIM27*, and *TOPORS* are critical regulators of SUMOylation: *TRIM27* and *TOPORS* function as E3 ligases (rate-limiting enzymes in SUMOylation), while *SENP6* acts as a SUMO protease that specifically cleaves polySUMO2/3 chains to regulate DNA repair, apoptosis, and senescence [22]. In this study, clinical sample analysis first revealed that *TOPORS* was significantly upregulated in aortic tissues of AD patients, whereas *SENP6* and *TRIM27* expression remained unchanged. BAPN-induced animal models further demonstrated that *TOPORS* expression correlated with medial aortic injury, inflammatory responses, and VSMC dysfunction. Notably, *TOPORS* inhibition in BAPN-induced AD mice significantly reduced aortic dilation, elastic fiber fragmentation, and serum inflammatory cytokine levels, suggesting *TOPORS* as a potential driver of AD progression.

*TOPORS* was initially identified as a cellular binding partner of DNA topoisomerase I and *p53*. Subsequent studies showed that *TOPORS* enhances SUMO-1 conjugation to *p53* in vitro and in vivo, with *TOPORS*-induced *p53* SUMOylation associated with increased endogenous p53 protein levels [23,24,25]. In AD pathogenesis, massive VSMC apoptosis critically weakens aortic wall integrity. As a classic pro-apoptotic transcription factor, *p53* is significantly upregulated in AD patients [26]. Consistent with this, our study found elevated *p53* mRNA/protein expression and apoptosis in AD mouse models and VSMC models, which were reversed by *TOPORS* silencing. These results indicate that aberrant *TOPORS* overexpression promotes VSMC apoptosis via enhanced *p53* SUMOylation during AD progression.

Inflammation is another key driver of AD. During AD progression, immune cell infiltration (e.g., macrophages, neutrophils) increases significantly [27], leading to excessive secretion of pro-inflammatory cytokines (*IL-1β*, *TNF-α*, *IL-6*) that activate VSMC apoptotic pathways, upregulate matrix metalloproteinases (MMPs), and exacerbate aortic wall damage [28]. Our study demonstrated that treating VSMC models with the *p53* inhibitor PFTα significantly reduced secretion of *IL-1β*, *TNF-α*, *IL-6*, and *IFN-*α, thereby alleviating AD pathogenesis. Combining previous findings with our results, we hypothesize that *TOPORS* modulates *p53* stability and transcriptional activity via SUMOylation, thereby regulating inflammatory responses in AD.

The *PI3K/AKT* signaling pathway is a central regulator of cell survival; its inactivation directly impairs VSMC proliferation and migration [29,30]. Clinical studies have shown reduced phosphorylated AKT (p-AKT) levels in AD aortic walls, accompanied by dephosphorylation of the pro-apoptotic protein Bad and activation of Caspase-3/9, leading to increased VSMC apoptosis [31]. In our experiments, *TOPORS* silencing promoted PI3K and AKT phosphorylation, whereas *TOPORS* overexpression decreased their phosphorylation. These data suggest that *TOPORS* exacerbates aortic injury not only by mediating *p53* SUMOylation but also by inhibiting *PI3K/AKT* pathway activation.

Although *TOPORS* shows potential as a driver of AD progression, several key challenges need to be tackled before these findings can be applied clinically. The results come from preclinical models and small-scale clinical sample analyses; large, multi-center clinical trials are necessary to confirm the link between *TOPORS* expression levels and AD severity, along with the safety and effectiveness of therapies targeting *TOPORS*. Moreover, the intricate interactions among *TOPORS*, *p53*, and the *PI3K/AKT* pathway imply that treatments solely targeting *TOPORS* might have restricted efficacy, emphasizing the demand for combination therapies that concurrently regulate multiple disease-related pathways. Concerning patient classification, our data indicate that non-syndromic AD patients, especially those with heightened *TOPORS* expression and *p53* overactivation in aortic tissues, are most apt to benefit from *TOPORS* suppression. Additionally, THE research offers a genetic screening tool for higher dissection risk.

## 5. Conclusions

In conclusion, this study is the first to demonstrate that *TOPORS* plays a critical role in the pathological progression of aortic dissection (AD) by regulating *PI3K/AKT*, the *p53* signaling pathway and inflammatory responses through SUMOylation. These findings not only expand the current understanding of AD’s molecular mechanisms but also identify *TOPORS* as a novel candidate target for AD therapy, potentially facilitating a paradigm shift in clinical strategy. Future research should address existing technical limitations to accelerate the clinical translation of *TOPORS*-related mechanisms, thereby providing theoretical support for reducing AD-associated mortality and disability rates.

## Figures and Tables

**Table 1:** Primer sequences of genes for qRT-PCR assay.

Genes	Sequence (5′-3′)
*β-Actin* (Human)	F: TCCTCCTGAGCGCAAGTACTCC
	R: CATACTCCTGCTTGCTGATCCAC
*TOPORS* (Human)	F: CGACACCGACCTAGCTTTCT
	R:CCTTAGCAGCTGATGCCATT
*SENP6* (Human)	F: CGGGTGCGGCCATTT
	R:GCCGTGGGTTCCCAAGA
*TRIM27* (Human)	F: GAGCAAATCCAGAACCAG
	R:TCAAACTCCCAAACAATC
*PI3K* (Human)	F: TTATAAACGAGAACGTGTG
	AATAGCTAGATAAGCC
*AKT* (Human)	F: CAGCATCGCTTCTTTGCCGGTA
	R:CCTGGTGTCAGTCTCCGACGTGA
*p53* (Human)	F: GGCCCATCCTCACCATCATCACA
	R:GCTCCCCTTTCTTGCGGAGA
*β-Actin* (Mouse)	CTCCTGAGCGCAAGTACTCT
	TACTCCTGCTTGCTGATCCAC
*TOPORS* (Mouse)	F: GCCTTCCTATAATGGTTCCT
	R:CGTGGTTGTTCTCCTACTT
*PI3K* (Mouse)	F: CGTGATGGAAAATATGGCTT
	R:TTGATCCTGCTGGTATTTGG
*AKT* (Mouse)	F: TGCCCTGGACTACTTGCACT
	R:ATCTTGATGTGCCCGTCCT
*p53* (Mouse)	F: AGACTCCAGTGGGAACCTT
	R:CTTCTTCTGTACGGCGGTCTC

## Data Availability

The datasets used and analyzed during the current study are available from the corresponding author on reasonable request.
